# Short-Term and Mid-Term Clinical Outcomes Following Hybrid Coronary Revascularization Versus Off-Pump Coronary Artery Bypass: A Meta-Analysis

**DOI:** 10.5935/abc.20180044

**Published:** 2018-04

**Authors:** Li Dong, Yi-kun Kang, An Xiang-guang

**Affiliations:** Heart Center & Beijing Key Laboratory of Hypertension - Beijing Chaoyang Hospital - Capital Medical University, Beijng - China

**Keywords:** Coronary Artery Disease/surgery, Coronary Artery Bypass, Off-Pump, Myocardial Revascularization/trends, Meta-Analysis, Database Bibliographic

## Abstract

**Background:**

Off-pump coronary artery bypass grafting (OPCAB) is one of the standard treatments for coronary artery disease (CAD) while hybrid coronary revascularization (HCR) represents an evolving revascularization strategy. However, the difference in outcomes between them remains unclear.

**Objective:**

We performed a meta-analysis to compare the short-term and mid-term outcomes of HCR versus OPCAB for the treatment of multivessel or left main CAD.

**Methods:**

We searched the PubMed, EMBASE, Web of Science and Cochrane databases to identify related studies and a routine meta-analysis was conducted.

**Results:**

Nine studies with 6121 patients were included in the analysis. There was no significant difference in short-term major adverse cardiac and cerebrovascular event (MACCE) rate (RR: 0.55, 95% CI: 0.30-1.03, p = 0.06) or mortality (RR: 0.51, 95% CI: 0.17-1.48, p = 0.22). HCR required less ventilator time (SMD: -0.36, 95% CI: -0.55- -0.16, p < 0.001), ICU stay (SMD: -0.35, 95% CI: -0.58 - -0.13, p < 0.01), hospital stay (SMD: -0.29, 95% CI: -0.50- -0.07, p < 0.05) and blood transfusion rate (RR: 0.57, 95% CI: 0.49-0.67, p < 0.001), but needed more operation time (SMD: 1.29, 95% CI: 0.54-2.05, p < 0.001) and hospitalization costs (SMD: 1.06, 95% CI: 0.45-1.66, p < 0.001). The HCR group had lower mid-term MACCE rate (RR: 0.49, 95% CI: 0.26-0.92, p < 0.05) but higher rate in mid-term target vessel revascularization (TVR, RR: 2.20, 95% CI: 1.32-3.67, p < 0.01).

**Conclusions:**

HCR had similar short-term mortality and morbidity comparing to OPCAB. HCR decreased the ventilator time, ICU stay, hospital stay, blood transfusion rate and increased operation time and hospitalization costs. HCR has a lower mid-term MACCE rate while OPCAB shows better in mid-term TVR.

## Introduction

Surgical revascularization still plays an essential role in the treatment of coronary artery disease (CAD) even in the era of widely prevalent percutaneous coronary intervention (PCI). As the most classical and widespread procedure for revascularization, coronary artery bypass grafting (CABG) has been considered the gold standard therapy in the past decades.^1^ In order to be safe and less disruptive, hybrid coronary revascularization (HCR) and off-pump coronary artery bypass grafting (OPCAB) which combines an off-pump technique with total arterial grafting. Recent years, more and more cardiac centers in the world have adopted OPCAB and HCR.^2,3^

It has been intensively discussed whether OPCAB is superior for CAD compared with on-pump CABG, but it remains uncertain. A recent randomized controlled trial (RCT) including 4752 patients found that the outcomes of death, stroke, myocardial infarction, renal failure or repeat revascularization at 5-year follow-up were similar among patients who underwent OPCAB or on-pump CABG.^4^ Another research investigated 3445 patents with a 13-year follow-up and drew conclusions that both OPCAB and on-pump CABG were safe and effective, and no significant difference was observed between them.^5^ However, a meta-analysis including 12 studies detected a lower rate of death and adverse effects after OPCAB compared with conventional CABG.^6^ Generally speaking, OPCAB is considered as lower incidence of neurological complications (including stroke, cognitive decline, etc.),^7^ in addition to a comparable less mortality and morbidity, particularly in high-risk groups and elderly patients.^8,9^

HCR combines minimally invasive CABG and PCI, offering a relatively atraumatic therapy for multivessel CAD. HCR utilizes a left internal mammary artery (LIMA) graft to the left anterior descending (LAD) coronary artery with drug-eluting stents (DES) to non-LAD target coronary arteries. Several studies have proved the excellent postoperative survival (higher than 99%) and LIMA patency rates (higher than 95%) of HCR, suggesting HCR should be considered as an alternative approach for patients with multivessel CAD.^10^ A study in France confirmed the feasibility and safety of HCR and also detected that HCR compared favorably to those with traditional CABG alone.^11^ In addition, both of simultaneous and staged HCR were indicated to be efficient and feasible with favorable outcomes at more than 12-month follow-up.^12,13^ However, a 1-year clinical follow-up study angiographically showed a high rate of repeat revascularization after HCR.^14^ In addition, a transient reduction in the antiplatelet effect of aspirin and clopidogrel was observed after HCR despite limited surgical trauma and off-pump technique.^15^ Neither baseline platelet aggregation nor postoperatively increased platelet turnover and acute-phase response could explain it. Therefore, further research is badly needed.

Currently, several comparative studies about the clinical outcomes of OPCAB and HCR are available. Nonetheless, the optimal surgical strategy remains disputable. In the present analysis, we sought to compare the short-term and mid-term clinical outcomes of HCR versus OPCAB for the treatment of multivessel or left main CAD with a pooled data.

## Methods

### Search strategy and selection criteria

We searched four electronic bibliographic databases including PubMed, EMBASE, Web of Science and Cochrane by using following keywords with different combinations: “coronary artery disease”, “multivessel coronary artery disease”, “left main coronary artery disease”, “no-touch coronary artery bypass”, “off-pump coronary artery bypass”, “hybrid coronary revascularization”, “minimally invasive coronary artery bypass” and “percutaneous coronary intervention”. The searches were limited to human studies and English-language literatures only. The last search date was March 1, 2017.

Inclusion criteria were: (1) RCTs, cohort studies or case-control trials (CCT) comparing the outcomes of HCR and OPCAB; (2) at least 15 participants in each group; (3) available to get complete data. In addition, exclusion criteria were: (1) duplicated papers that fail to provide supplementary information; (2) unfinished studies or unavailable data (3) studies with obvious defects in design or data statistics. Two researchers selected literatures and any disagreements were resolved through consensus.

### Data extraction and quality assessment

For articles approved in the primary selection, two reviewers assessed the quality of studies and extract data independently. The CONSORT statement^16^ and STROBE statement^17^ were used to measure the quality of RCTs and observational studies, respectively. Low-quality studies should be excluded and any disagreements were resolved by consensus or judged by the senior author.

Extracted information included: (1) characteristics of studies and patients; (2) basic management of HCR and OPCAB; (3) short-term (in-hospital or 30-day) and mid-term (3 months to 36 months) mortality, stroke and major adverse cardiac and cerebrovascular event (MACCE) which was defined as the incidence of all-cause death, stroke, myocardial infarction (MI) and target vessel revascularization (TVR); (4) in-hospital outcomes: operation time, ventilator time, ICU stay, hospital stay, blood transfusion rate, incidence of atrial fibrillation (AF) and hospitalization costs.

### Statistical analysis

We performed the analyses using RevMan 5.3 software (Cochrane Collaboration, Copenhagen, Denmark). Relative risk (RR) with 95% confidence interval (CI) was calculated for dichotomous variables and standardized mean difference (SMD) with 95% CI was calculated for continuous variables. Then *Forest plots* were presented graphically for all clinical outcomes. Statistical heterogeneity between studies was calculated using chi-squared test and the *I*-squared measure on a scale of 0-100% (less than 50% represented a low heterogeneity, 50%-75% indicated a moderate inconsistency and higher than 75% meant a large degree of heterogeneity). Fix-effect model was used in analysis with heterogeneity < 50% while random-effect model was conducted with heterogeneity ≥ 50%. In addition, publication bias of short-term (in-hospital or 30-day) MACCE rate was also assessed using funnel plot. Two-sided p value < 0.05 was considered statistically significant.

## Results

### Literature selection and characteristics of studies

**Figure 1:**
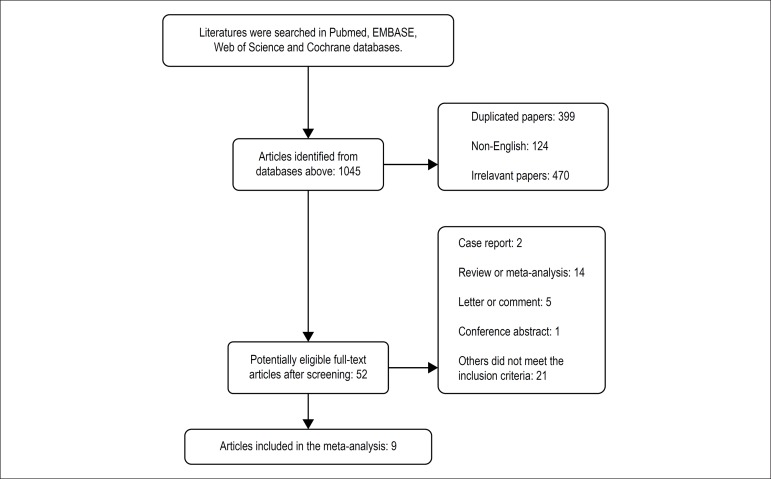
Flow diagram shows the process of literature selection.

The process of literature selection for potentially eligible studies and exclusion reasons is illustrated using a flow diagram in Figure 1. Initially, 1045 published articles were identified (455 from PubMed, 469 from EMBASE, 106 from Web of Science and 15 from Cochrane). Overall, 52 unduplicated English articles related to HCR and OPCAB were selected from these citations. Finally, nine observational studies with 6121 patients were included in the present analysis.^18-26^

The basic characteristics of these studies are presented in Table 1. Among 6121 patients, 5418 (88.5%) subjects got OPCAB while 290 (4.7%) patients received staged HCR and 398 (6.7%) patients received simultaneous HCR. For those who underwent HCR, minimal invasive techniques such as endoscopic atraumatic coronary artery bypass (endo-ACAB), mini-sternotomy and mini-thoracotomy were utilized. Most of them received DES and a combination of aspirin and clopidogrel was applied as a preventive antiplatelet therapy. Short-term (in-hospital or 30-day) and mid-term clinical outcomes are shown in Table 2.

**Table 1:** Characteristics of the included studies

References	Year	Primary endpoint	Follow-up	HCR							OPCAB		
				Number of patients	Mean age	Baseline LVEF (%)	Setting	Surgery type	Stents	Antiplatelet strategy	Number of patients	Mean age	Baseline LVEF (%)
Kon^18^	2008	In-hospital MACCE	1 year	15	61.0 ± 10.0	47.0 ± 14.0	Simultaneous	Smallthoracotomy	DES	Aspirin 325 mg, clopidogrel 300 mg	30	65.0 ± 10.0	45.0 ± 14.0
Vassiliades^19^	2009	In-hospital mortality	1 year	91	64.7 ± 13.7	51.5 ± 9.4	Staged	Endo-ACAB	DES(85.8%)	Aspirin 81-162 mg, clopidogrel 75 mg	4175	62.8 ± 11.7	50.9 ± 12.7
Hu^20^	2010	In-hospital MACCE	Mean 18 months	104	61.8 ± 10.2	62.4 ± 6.9	Simultaneous	Ministernotomy	DES	Aspirin 100 mg, clopidogrel 300 mg	104	62.4 ± 8.0	63.4 ± 7.5
Halkos^21^	2011	In-hospital MACCE	Median 3.2 years	147	64.3 ± 12.8	54.6 ± 8.7	Staged	Endo-ACAB with robotic assistance	DES (mojority)	Clopidogrel 600 mg	588	64.3 ± 12.5	54.7 ± 8.7
Halkos^22^	2011	In-hospital and 30-day MACCE	Median 3.2 years	27	63.9 ± 13.7	56.6 ± 7.7	Staged	Mini-sternotomy, robotic assistance	DES(92.6%)	Clopidogrel 600 mg	81	63.9 ± 12.7	56.6 ± 7.6
Bachinsky^23^	2012	In-hospital and 30-day MACCE	30 days	25	63.2 ± 10.5	55.3 ± 10.4	Staged	Thoracotomy with robotic assistance	DES(71.0%)	Aspirin 325 mg, clopidogrel 600 mg	27	66.8 ± 10.7	51.5 ± 12.0
Zhou^24^	2013	In-hospital MACCE	30 days	141	62.0 ± 10.1	61.8 ± 6.9	Simultaneous	Mini-sternotomy	DES	Aspirin 100 mg, heparin 120 IU/kg	141	63.2 ± 8.5	60.1 ± 9.3
Harskamp^25^	2014	cTnI after 24h	1 year	33	65.0 ± 6.5	55.0 ± 7.5	Simultaneous	Mini-thoracotomy with robotic assistance	DES(75.8%)	Aspirin and clopidogrel	32	67.0 ± 7.0	55.0 ± 5.0
Song^26^	2016	In-hospital outcomes	Median 2.5 years	120	62.3 ± 9.4	63.9 ± 7.3	Simultaneous	Mini-sternotomy	DES(99.5%)	Aspirin 100 mg, clopidogrel 300 mg	240	62.8 ± 8.4	64.2 ± 6.9

MACCE: major adverse cardiac and cerebrovascular event, cTnI: cardiac troponin I, HCR: hybrid coronary revascularization, OPCAB: Off-pump coronary artery bypass grafting, LVEF: left ventricular ejection fraction, endo‑ACAB: endoscopic atraumatic coronary artery bypass, DES: drug-eluting stent.

**Table 2:** Short-term and mid-term clinical outcomes of the included studies

References	Time of outcomes	HCR						OPCAB					
		Number of patients	MACCE	Death	Stroke	MI	TVR	Number of patients	MACCE	Death	Stroke	MI	TVR
Kon^18^	Short-term	15	0	0	0	0	0	30	7	0	1	6	0
	Mid-term	15	1	0	0	0	1	30	7	0	0	0	0
Vassiliades^19^	Short-term	91	1	0	1	0	0	4175	126	74	47	20	12
	Mid-term	91	10	1	1	1	7	4175	--	230	--	--	--
Hu^20^	Short-term	104	0	0	0	0	0	104	0	0	0	0	0
	Mid-term	104	1	0	0	0	1	104	10	1	5	0	3
Halkos^21^	Short-term	147	3	1	1	1	0	588	12	5	4	3	0
	Mid-term	147	--	--	--	--	13	588	--	--	--	--	18
Halkos^22^	Short-term	27	0	0	0	0	0	81	4	3	0	2	0
	Mid-term	27	--	--	--	--	2	81	--	--	--	--	1
Bachinsky^23^	Short-term	25	0	0	0	0	0	27	1	1	0	0	0
Zhou^24^	Short-term	141	7	1	1	5	0	141	10	2	1	7	0
Harskamp^25^	Short-term	33	1	1	0	0	0	32	1	1	0	0	0
	Mid-term	33	1	1	0	0	2	32	2	1	0	1	1
Song^26^	Mid-term	120	8	3	0	0	5	237	19	6	8	2	6

HCR: hybrid coronary revascularization, OPCAB: Off-pump coronary artery bypass grafting, MACCE: major adverse cardiac and cerebrovascular event, MI: myocardial infarction, TVR: target vessel revascularization.

### Short-term outcomes

As illustrated in Table 3, there was no significant difference in short-term MACCE rate (relative risk (RR): 0.55, 95% confidence interval (CI): 0.30-1.03, p = 0.06; p for heterogeneity = 0.85, I^2^ = 0%) or mortality (RR: 0.51, 95% CI: 0.17-1.48, p = 0.22; p for heterogeneity = 0.99, I^2^= 0%) or stroke (RR: 0.93, 95% CI: 0.28-3.05, p = 0.90; p for heterogeneity = 1.00, I^2^ = 0%) between the two groups. HCR required less ventilator time (standardized mean difference (SMD): -0.36, 95% CI: -0.55- -0.16, p < 0.001), ICU stay (SMD: -0.35, 95% CI: -0.58- -0.13, p < 0.01), hospital stay (SMD: -0.29, 95% CI: -0.50- -0.07, p < 0.05) and blood transfusion rate (relative risk (RR): 0.57, 95% CI: 0.49-0.67, p < 0.001), but needed more operation time (SMD: 1.29, 95% CI: 0.54-2.05, p < 0.001) and hospitalization costs (SMD: 1.06, 95% CI: 0.45-1.66, p < 0.001).

**Table 3:** Summary of results for short-term clinical outcomes of HCR versus OPCAB

Outcomes	Number of studies	Total numbers of patients	SMD or RR	95% CI	p value
Short-term MACCE rate	8	5761	0.55	[0.30, 1.03]	0.06
Staged HCR	4	5161	0.58	[0.23, 1.47]	0.25
Simultaneous HCR	4	600	0.54	[0.23, 1.23]	0.14
Short-term mortality	8	5761	0.51	[0.17, 1.48]	0.22
Staged HCR	4	5161	0.46	[0.12, 1.73]	0.25
Simultaneous HCR	4	600	0.66	[0.11, 3.88]	0.64
Short-term stroke	8	5761	0.93	[0.28, 3.05]	0.90
Operation time	3	542	1.29	[0.54, 2.05]	< 0.001
Ventilator time	6	1861	-0.36	[-0.55, -0.16]	< 0.001
ICU stay	7	1913	-0.35	[-0.58, -0.13]	0.002
Hospital stay	7	1538	-0.29	[-0.50, -0.07]	0.01
Blood transfusion rate	6	1361	0.57	[0.49, 0.67]	< 0.001
AF rate	7	1933	1.08	[0.83, 1.40]	0.56
Hospitalization costs	3	305	1.06	[0.45, 1.66]	< 0.001

HCR: hybrid coronary revascularization, OPCAB: Off-pump coronary artery bypass grafting, MACCE: major adverse cardiac and cerebrovascular event, AF: atrial fibrillation, SMD: standardized mean difference, RR: relative risk, CI: confidence interval.

### Subgroup analysis

Table 3 also showed the subgroup analysis, which was performed by dividing the studies into staged-HCR group and simultaneous-HCR group. No statistical difference was observed in short-term MACCE rate or mortality in the two subgroups (*P* value in both subgroups > 0.05).

### Mid-term outcomes

**Figure 2:**
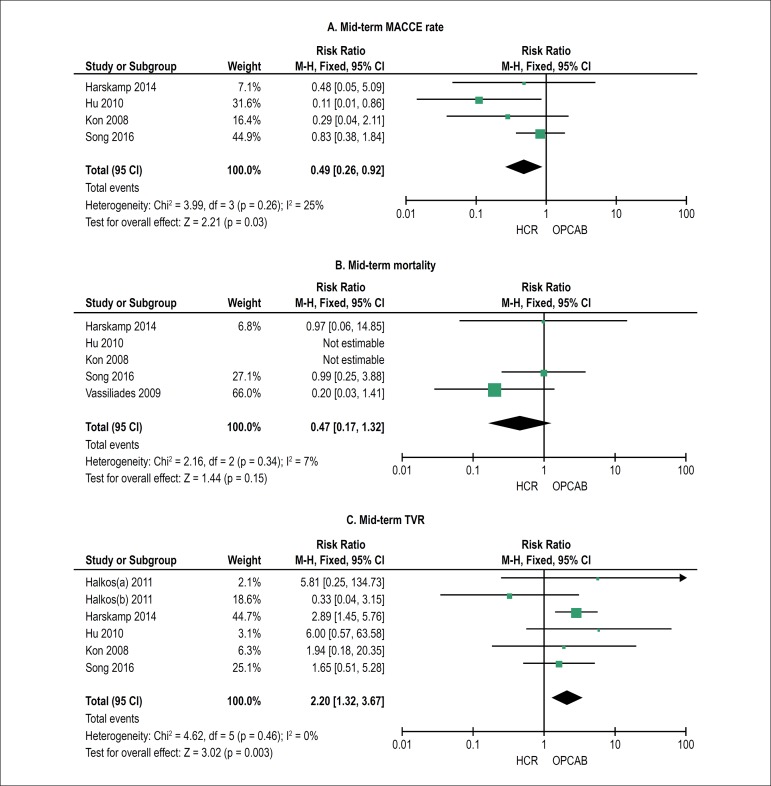
Meta-analysis shows the relative risk (RR) of mid-term MACCE rate, mortality and TVR. MACCE: major adverse cardiac and cerebrovascular event, TVR: target vessel revascularization, CI: confidence interval, HCR: hybrid coronary revascularization; OPCAB: off-pump coronary artery bypass grafting.

The studies that contained mid-term outcomes were included in the analysis. As shown in Figure 2, the HCR group had lower MACCE rate (RR: 0.49, 95% CI: 0.26-0.92, p < 0.05, P for heterogeneity = 0.26, I^2^ = 25%) but had higher rate in TVR (RR: 2.20, 95% CI: 1.32-3.67, p < 0.01, P for heterogeneity = 0.46, I^2^ = 0%) in mid-term follow. No significant difference in mid-term mortality was detected between the two groups (RR: 0.47, 95% CI: 0.17-1.32, p < 0.01, P for heterogeneity = 0.34, I^2^ = 7%).

### Heterogeneity

In the current analysis, no obvious heterogeneity was found between studies in either short-term or mid-term MACCE rate and mortality (p for heterogeneity > 0.05, I^2^< 50%). And subgroup analysis showed no heterogeneity (p for heterogeneity = 0.95, I^2^ = 0%).

### Publication bias

**Figure 3:**
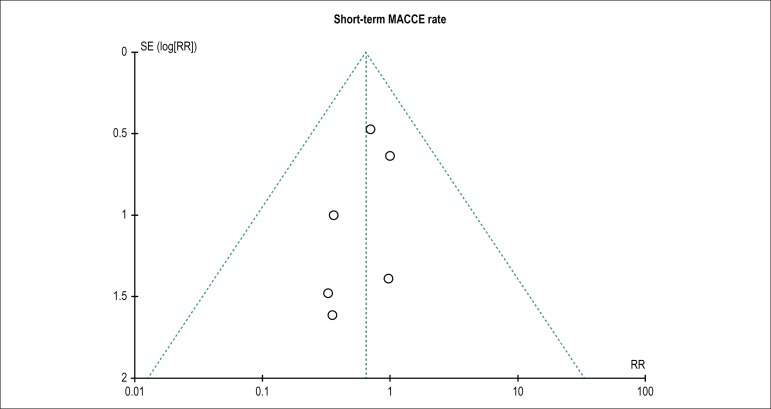
Funnel plot shows the test for publication bias of short-term (in-hospital or 30-day) mortality and MACCE rate. MACCE: major adverse cardiac and cerebrovascular event, RR: relative risk, SE: standard error.

The funnel graph of short-term MACCE rate was established in Figure 3, and there was no evident publication bias among all included studies by visual examination.

## Discussion

The present meta-analysis shows that HCR, compared with OPCAB, seems not to significantly improve short-term mortality and morbidity of postoperative complications for patients with CAD. These results are similar to previews research. Hu^27^ first systematically compared the short-term clinical outcomes after HCR versus OPCAB for the treatment of multivessel or left main CAD, and most of the results were consistent with the current analysis. However, some differences between the two analyses should be also mentioned. We excluded one study^28^ due to small sample size(less than 15 patients), outdated surgical procedures (8-10 cm thoracotomy incisions), different kinds of DES (cypher or taxus), uncertainty of baseline LVEF (not reported) and high heterogeneity in analysis. We also put three recent high-quality studies into pooled data so that all outcomes are updated. In addition, in the present study, we focus on postoperative complications and take stroke as a primary endpoint. Therefore, the present analysis is needed for a better elucidation of HCR and OPCAB.

To our knowledge, this is the first meta-analysis comparing the mid-term clinical outcomes between HCR and OPCAB so far. Our data shows that HCR has a lower mid-term MACCE rate while OPCAB shows a better result in mid-term TVR. Moreover, no significant difference in mid-term mortality was detected between the two groups. Patients undergoing the hybrid procedure have relatively better mid-term clinical outcomes probably owing to reduced myocardial manipulation and activation of coagulation^26^^.^ It has been widely recognized that the dislodgement or rupture of atherosclerotic plaques during surgical aortic manipulation results in a major cause of stroke.^29^ Since the aorta is more or less affected in the surgical procedure, it is still unclear whether OPCAB can decrease postoperative stroke rate compared with on-pump CABG. In contrast, grafting in HCR only involves LAD artery while other coronary arteries are treated by PCI. As a result, low rate of neurological complications becomes one of the main advantages of HCR. Although, in the present analysis we detect no significant difference of stroke rate between OPCAB and HCR in a short-term follow-up, which seems to be contradictory to some previous analyses.

However, Song et al.^26^ reported that more patients in OPCAB group suffer from stroke than HCR group in a 30-month follow-up, which indicates that the differences may be well recognized in a long-term follow-up. In recent years, technical advances in OPCAB utilize a no-touch technique to avoid aortic manipulation during grafting. A retrospective study showed that the OPCAB with no-touch technique could improve prognosis by minimizing the neurological complications and the morbidity.^30^ Emmert et al.^31^ also reported that the aortic no-touch OPCAB provided superior neurological outcomes than on-pump CABG and no-touch technique should be properly applied. Halbersma et al.^32^ investigated the four-year clinical outcomes after OPCAB with no-touch technique and concluded that it was a safe and efficient choice for patients with multivessel or left main CAD. Compelling data have indicated that the combination of OPCAB and clampless strategies can reduce stroke risk. However, the major shortcoming of no-touch OPCAB is its greater technical requirement so that it is not applicable for every surgical team or every patient.^33^ Nevertheless, further investigations should be still carried out to compare no-touch OPCAB and HCR.

In the current analysis, neither staged HCR nor simultaneous HCR makes a difference to the short-term outcomes, which is consistent with former studies.^27^ Commonly, there are three strategies for HCR: (1) performing LIMA-LAD grafting first and then followed by PCI, the interval varies from several hours to a few weeks; (2) vice versa; (3) combined LIMA-LAD grafting and PCI at the same time in a hybrid operative unit. The optimal sequence of LIMA-LAD grafting and PCI has been debated but still remains unclear. In fact, most centers choose their own surgical procedures mainly based on preferences of physicians, considerations of patients, economic issues and available resources. Although several studies have indicated that both simultaneous and staged HCR contribute to excellent results, most centers prefer to adopt the latter one with LIMA-LAD grafting performed first.^34^ The CABG-first approach is recommended by the American College of Cardiology Foundation/American Heart Association^35^ and it has some obvious advantages. It can reduce the overlapping from two different teams so that they can perform in their most familiar way and avoid to interacting with each other in operation room. Then antiplatelet and antithrombotic strategies can be well managed and adjusted according to physicians from different teams.^36^ However, the disadvantages include that patients have to undergo at least two surgeries and need more time to recover. Moreover, hemorrhagic tendency and overload of kidneys also deserve significant attention. Currently, no study has compared the clinical outcomes of staged HCR and simultaneous HCR directly, so further research should be placed on it.

In the present analysis, we also confirm that HCR apparently decreases the ventilator time, ICU stay, hospital stay and blood transfusion rate comparing to OPCAB. Although these items may not directly influence the main outcomes, they are also important criteria to judge a surgical procedure. Several reasons may account for these advantages of HCR. With the development of surgical procedures, endoscopic technique and mini incision are widely utilized in HCR to help patients ease suffering and recover sooner.^37^ And retractor-stabilizer, such as robot, provides access that LIMA-LAD grafting can be performed with accuracy and precision with minimally invasive thoracotomy or sternotomy.^38^ Practically, with the assistance of a surgical robot, it offers an excellent visual field and reduces operation time. However, some drawbacks of HCR also deserve our attention. Our study detects that the hybrid procedure required longer operation time and incurred much higher in-hospital costs than OPCAB. In Bachinsky`s study,^23^ despite lower postoperative costs, the HCR group still needs more overall hospital costs owning to its higher procedural costs. Consequently, pros and cons of HCR should be weighed and considered carefully before operation.

Some limitations of the present analysis should be also emphasized. Firstly, all included studies belong to observational studies and no single RCT has been conducted so far. Secondly, some included studies contain relatively small samples (fewer than 50 patients) and remain imbalance of patient number between groups so that deviation of results may inevitably exist. Thirdly, long-term patency is more convincing than short-term and mid-term outcomes, but very limited references were published with long-term follow-up so far. Finally, some uncontrolled factors may interfere with the current analysis. Variables like gender ratio and LVEF at baseline have not been adjusted. And diverse surgery procedures, stents (DES or bare stent) as well as antiplatelet strategies may disturb the accuracy of results too.

## Conclusions

HCR shows similar results with OPCAB in short-term clinical outcomes. HCR decreases the ventilator time, ICU stay, hospital stay, blood transfusion rate and increases the operation time and hospitalization costs. Although repeated vessel revascularization is greater with HCR, it has a lower mid-term MACCE rate and could provide a safe and reproducible alternative for patients with multivessel CAD.

## References

[1] Ennker JC, Ennker IC (2012). Coronary artery surgery: now and in the next
decade. HSR Proc Intensive Care and Cardiovasc Anesth.

[2] Arom KV, Flavin TF, Emery RW, Kshettry VR, Petersen RJ, Janey PA (2000). Is low ejection fraction safe for off-pump coronary artery bypass
operation. Ann Thorac Surg.

[3] Gao C, Yang M, Wu Y, Wang G, Xiao C, Liu H (2009). Hybrid coronary revascularization by endoscopic robotic coronary
artery bypass grafting on beating heart and stent placement. Ann Thorac Surg.

[4] Lamy A, Devereaux PJ, Prabhakaran D, Taggart DP, Hu S, Straka Z, CORONARY Investigators (2016). Five-year outcomes after off-pump or on-pump coronary-artery
bypass grafting. N Engl J Med.

[5] Riess FC, Heller S, Cramer E, Awwad N, Amin W, Hansen L (2016). On-pump versus off-pump complete arterial revascularization using
bilateral internal mammary arteries and the t-graft technique clinical and
angiographic results for 3,445 patients in 13 years of
follow-up. Cardiology.

[6] Sepehripour AH, Chaudhry UA, Suliman A, Kidher E, Sayani N, Ashrafian H (2016). How revascularization on the beating heart with cardiopulmonary
bypass compares to off-pump A meta-analysis of observational
studies. Interact Cardiovasc Thorac Surg.

[7] Vallely MP, Potger K, McMillan D, Hemli JM, Brady PW, Brereton RJ (2008). Anaortic techniques reduce neurological morbidity after off-pump
coronary artery bypass surgery. Heart Lung Circ.

[8] Lemma GM, Coscioni E, Centofanti P, Centofanti P, Fondacone C, Salica A (2012). On-pump versus off-pump coronary artery bypass surgery in
high-risk patients: operative results of a prospective randomized trial
(on-off study). J Thorac Cardiovasc Surg.

[9] Falk V (2010). Stay off-pump and do not touch the aorta!. Eur Heart J.

[10] Bonatti JO, Zimrin D, Lehr EJ, Vesely M, Kon ZN, Wehman B (2012). Hybrid coronary revascularization using robotic totally
endoscopic surgery: perioperative outcomes and 5-year
results. Ann Thorac Surg.

[11] Delhaye C, Sudre A, Lemesle G, Vanesson L, Koussa M, Fayad G (2010). Hybrid revascularization, comprising coronary artery bypass graft
with exclusive arterial conduits followed by early drug-eluting stent
implantation, in multivessel coronary artery disease. Arch Cardiovasc Dis.

[12] Adams C, Burns DJ, Chu MW, Jones PM, Shridar K, Teefy P (2014). Single-stage hybrid coronary revascularization with long-term
follow-up. Eur J Cardiothorac Surg.

[13] Versaci F, Reimers B, Del Giudice C, Schofer J, Giacomin A, Saccà S (2009). Simultaneous hybrid revascularization by carotid stenting and
coronary artery bypass grafting: the SHARP study. JACC Cardiovasc Interv.

[14] Modrau IS, Holm NR, Maeng M, Bøtker HE, Christiansen EH, Kristensen SD (2015). One-year clinical and angiographic results of hybrid coronary
revascularization. J Thorac Cardiovasc Surg.

[15] Modrau IS, Wurtz M, Kristensen SD, Hvas AM (2015). Reduced effect of aspirin and clopidogrel following hybrid
coronary revascularization. Clin Appl Thromb Hemost.

[16] Schulz KF, Altman DG, Moher D, CONSORT Group (2010). CONSORT 2010 Statement: updated guidelines for reporting parallel
group randomised trials. PLoS Med.

[17] Vandenbroucke JP, Von Elm E, Altman DG, Egger M, Gøtzsche PC, Mulrow CD, STROBE Initiative (2007). The strengthening the reporting of observational studies in
epidemiology (STROBE) statement: guidelines for reporting observational
studies. PLoS Med.

[18] Kon ZN, Brown EN, Tran R, Joshi A, Reicher B, Grant MC (2008). Simultaneous hybrid coronary revascularization reduces
postoperative morbidity compared with results from conventional off-pump
coronary artery bypass. J Thorac Cardiovasc Surg.

[19] Vassiliades TA, Kilgo PD, Douglas JS, Babaliaros VC, Block PC, Samady H (2009). Clinical outcomes after hybrid coronary revascularization versus
off pump coronary artery bypass. Innovations (Phila).

[20] Hu S, Li Q, Gao P, Xiong H, Zheng Z, Li L (2011). Simultaneous hybrid revascularization versus off-pump coronary
artery bypass for multivessel coronary artery disease. Ann Thorac Surg.

[21] Halkos ME, Vassiliades TA, Douglas JS, Morris DC, Rab ST, Liberman HA (2011). Hybrid coronary revascularization versus off-pump coronary artery
bypass grafting for the treatment of multivessel coronary artery
disease. Ann Thorac Surg.

[22] Halkos ME, Rab ST, Vassiliades TA, Morris DC, Douglas JS, Kilgo PD (2011). Hybrid coronary revascularization versus off-pump coronary artery
bypass for the treatment of left main coronary stenosis. Ann Thorac Surg.

[23] Bachinsky WB, Abdelsalam M, Boga G, Kiljanek L, Mumtaz M, McCarty C (2012). Comparative study of same sitting hybrid coronary artery
revascularization versus off-pump coronary artery bypass in multivessel
coronary artery disease. J Interv Cardiol.

[24] Zhou S, Fang Z, Xiong H, Hu S, Xu B, Chen L, Wang W (2014). Effect of one-stop hybrid coronary revascularization on
postoperative renal function and bleeding: a comparison study with off-pump
coronary artery bypass grafting surgery. J Thorac Cardiovasc Surg.

[25] Harskamp RE, Abdelsalam M, Lopes RD, Boga G, Hirji S, Krishnan M (2014). Cardiac troponin release following hybrid coronary
revascularization versus off-pump coronary artery bypass
surgery. Interact Cardiovasc Thorac Surg.

[26] Song Z, Shen L, Zheng Z, Xu B, Xiong H, Li L (2016). One-stop hybrid coronary revascularization versus off-pump
coronary artery bypass in patients with diabetes mellitus. J Thorac Cardiovasc Surg.

[27] Hu FB, Cui LQ (2015). Short-term clinical outcomes after hybrid coronary
revascularization versus off-pump coronary artery bypass for the treatment
of multivessel or left main coronary artery disease: a
meta-analysis. Coron Artery Dis.

[28] Reicher B, Poston RS, Mehra MR, Joshi A, Odonkor P, Kon Z (2008). Simultaneous 'hybrid' percutaneous coronary intervention and
minimally invasive surgical bypass grafting: feasibility, safety, and
clinical outcomes. Am Heart J.

[29] Lev-Ran O, Braunstein R, Sharony R, Kramer A, Paz Y, Mohr R, Uretzky G (2005). No-touch aorta off-pump coronary surgery: the effect on
stroke. J Thorac Cardiovasc Surg.

[30] Arrigoni SC, Mecozzi G, Grandjean JG, Hillege JL, Kappetein AP, Mariani MA (2015). Off-pump no-touch technique: 3-year results compared with the
SYNTAX trial. Interact Cardiovasc Thorac Surg.

[31] Emmert MY, Seifert B, Wilhelm M, Grünenfelder J, Falk V, Salzberg SP (2011). Aortic no-touch technique makes the difference in off-pump
coronary artery bypass grafting. J Thorac Cardiovasc Surg.

[32] Halbersma WB, Arrigoni SC, Mecozzi G, Grandjean JG, Kappetein AP, van der Palen J (2009). Four-year outcome of OPCAB no-touch with total arterial Y-graft:
making the best treatment a daily practice. Ann Thorac Surg.

[33] Yanagawa B, Nedadur R, Puskas JD (2016). The future of off-pump coronary artery bypass grafting: a North
American perspective. J Thorac Dis.

[34] Zhang L, Cui Z, Song Z, Yang H, Fu Y, Gong Y (2016). Minimally invasive direct coronary artery bypass for left
anterior descending artery revascularization - analysis of 300
cases. Wideochir Inne Tech Maloinwazyjne.

[35] Hillis LD, Smith PK, Anderson JL, Bittl JA, Bridges CR, Byrne JG, American College of Cardiology Foundation, American Heart Association Task Force on Practice
Guidelines (2012). 2011 ACCF/AHA guideline for coronary artery bypass graft surgery:
executive summary: a report of the American College of Cardiology
Foundation/American Heart Association Task Force on Practice
Guidelines. J Thorac Cardiovasc Surg.

[36] Halkos ME, Walker PF, Vassiliades TA, Douglas JS, Devireddy C, Guyton RA (2014). Clinical and angiographic results after hybrid coronary
revascularization. Ann Thorac Surg.

[37] Aubin H, Akhyari P, Lichtenberg A, Albert A (2015). Additional right-sided upper "Half-Mini-Thoracotomy" for
aortocoronary bypass grafting during minimally invasive multivessel
revascularization. J Cardiothorac Surg.

[38] Ejiofor JI, Leacche M, Byrne JG (2015). Robotic CABG and hybrid approaches: the current
landscape. Prog Cardiovasc Dis.

